# An intuitive explanation of dermoscopic structures by digitally reconstructed pathological horizontal top-down view images

**DOI:** 10.1038/s41598-019-56522-8

**Published:** 2019-12-27

**Authors:** Akira Kasuya, Masahiro Aoshima, Kensuke Fukuchi, Takatoshi Shimauchi, Toshiharu Fujiyama, Yoshiki Tokura

**Affiliations:** Department of Dermatology, Hamamatsu University School of Medicine, 1-20-1 Handayama, Higashi-Ku, Hamamatsu 431-3192 Japan

**Keywords:** Cancer screening, Skin manifestations

## Abstract

Dermoscopy is a convenient tool to diagnose melanocytic lesions, especially nevus and melanoma. Various pigmented structures, including pigment network, dots and globules, and streaks, are observed in dermoscopy. Usually, 2D vertical images are used to explain the correlation of dermoscopy and histopathology. However, because the image of dermoscopy is horizontal, it is difficult for the horizontal view of dermoscopy to refer to the vertical view of histopathology. In our study, we digitally reconstructed 2D horizontal top-down view images and 3D aerial images from 50–100 serial 2D vertical sections by using high-speed scanner and 3D software in 6 cases of melanocytic lesion. Our new technology intuitively explained the histopathological structures corresponding to the dermoscopic structures. This technique could be used as a good educational tool for beginners.

## Introduction

Dermoscopy is an optical observation device, by which we examine the detail of minute structures of melanocytic skin lesions. Clinicians usually use dermoscopy to make a diagnosis of benign nevi and malignant melanoma. In dermoscopic observations for melanocytic lesions, colored structures such as pigment network, dots & globules, and streaks are quite important. When these structures look like irregular in shape, color, and largeness, we infer a diagnosis of melanoma. Usually, the dermatoscopic-histologic correlation was analyzed with plane vertical images of pathology^1–8^, and therefore, the explanation is not greatly intuitive or forthcoming for beginners. Therefore, to provide an intuitive introduction for the dermatoscopic-histologic correlation, we implemented a three dimensional (3D) structure-based analysis and reconstructed horizontal images using serial sections of pigmented lesions.

In our previous study we have already successfully shown the 3D reconstructed images and horizontal images of regular pigment network^9^. Thus in this study we shows other structures such as irregular piment networks, pseudonetwork, dots, globules including cobble stone pattern, and streak. Furthermore we showed some hidden pathological structure of darkly homogeneously pigmented lesion by analyzing reconstructed horizontal images.

## Material and Method

### Dermoscopy and surgical specimen

Melanocytic skin lesions were examined with a dermoscope (DermLite DL3N, 3Gen inc, San Juan Capistrano, CA)^9^. Dermoscopic terminology was used according to the consensus^10^. Human skin surgical specimens were obtained from the archives of Hamamatsu University School of Medicine, and patients were kept anonymous. Our study is a retrospective observational study. Informed consent was done by the opt-out method of our hospital website as our previous study^9^. We obtained an approval from “Ethical committee of Hamamatsu University School of Medicine” (IRB 18-082) for the performance of our study. We implemented our experiment following the Helsinki declaration guidelines.

### Histological examination and immunohistochemistry

Fixing of surgical resected specimen was done in 3.5% paraformaldehyde and embedded in paraffin. Slices with a thickness of 5micro meter were stained with Fontana-Masson (FM, Melanin Stain). Hematoxylin staining was not performed, because the staining obscures the color of melanin.

### Reconstruction of 3D images

Fifty to 100 serial sections for each lesion were scanned by a digital scanner (Nano Zoomer, Hamamatsu photonics, Hamamatsu, Japan) as our previous study^9^. The scanned 2D images were used to reconstruct 3D image by a 3D visualization software, Voloom, (Micro Dimensions, Munich, Germany)^9,11,12^. Voloom is an automated software for 3D alignment enabling examination of tissue slices as a 3D volume.

## Result

### Clinical findings

**Figure 1:**
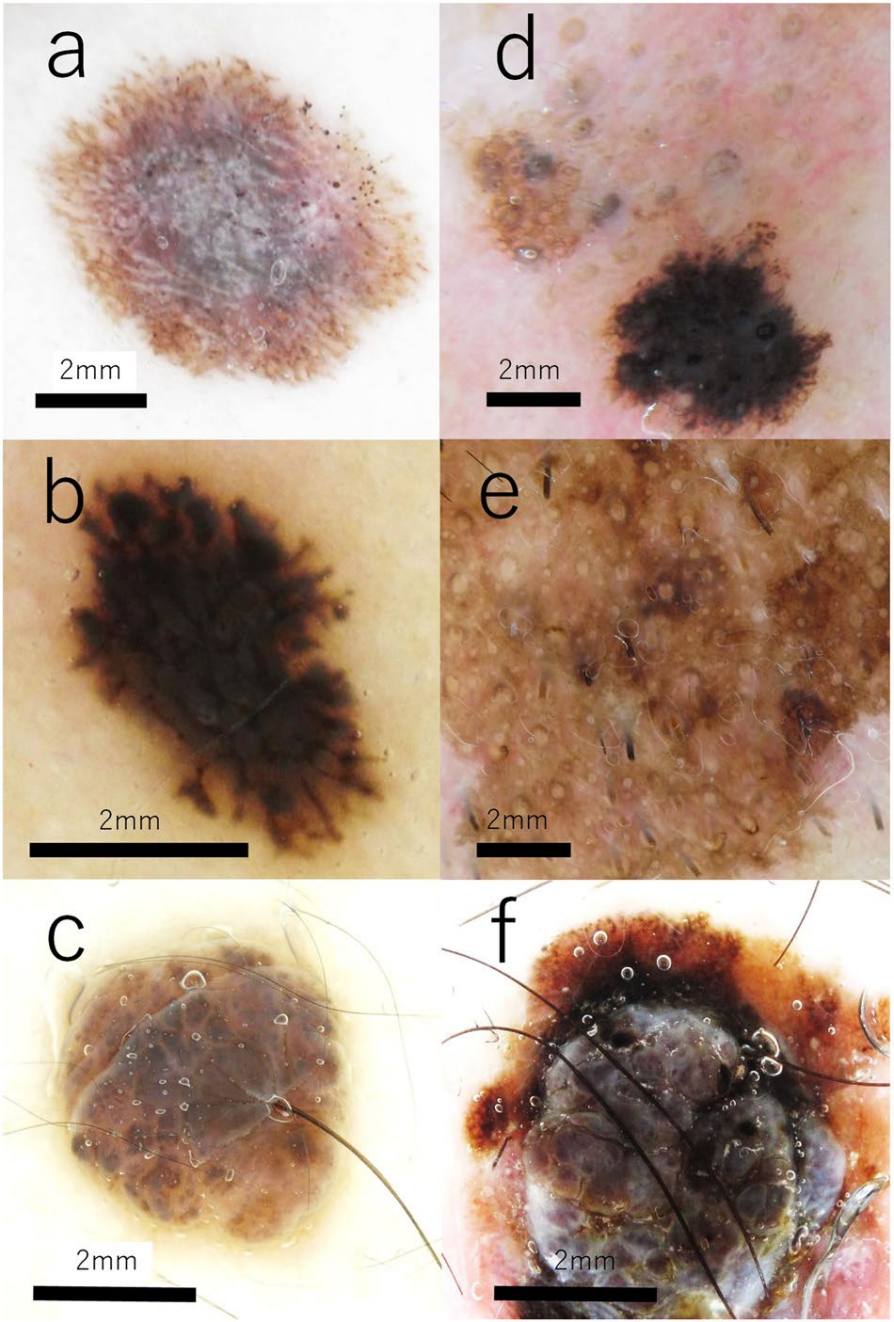
Dermoscopic images of pigmented lesions. (**a**) Nevus1, Spitz Nevus, 7 y.o. male, lower leg, (**b**) Nevus2, Reed nevus, 2 y.o. female, upper limb, (**c**) Nevus3, Miescher nevus, 39 y.o. female, back, (**d**) lentigo maligna melanoma (LMM) 1, 65 y.o. male, face, (**e**) LMM2, 75 y.o. male, face, (**i**) superficial spreading melanoma (SSM), 49 y.o. female, lower leg, Scale bars 2 mm.

A total of 6 pigmented skin lesions were observed in this study. The lesions included 1 Spitz nevus, 1 Reed nevus, 1 Miescher nevus, 2 lentigo maligna melanoma (LMM; LMM1 and 2) and 1 superficial spreading melanoma (SSM). Quantities of serial sections were 100 in Miescher nevus, LMM2 and SSM, 70 in Spitz nevus and LMM1, and 50 in Reed nevus, respectively. The dermoscopic images of pigmented lesions were shown in Fig. 1.

## Discussion

### Irregular network

**Figure 2:**
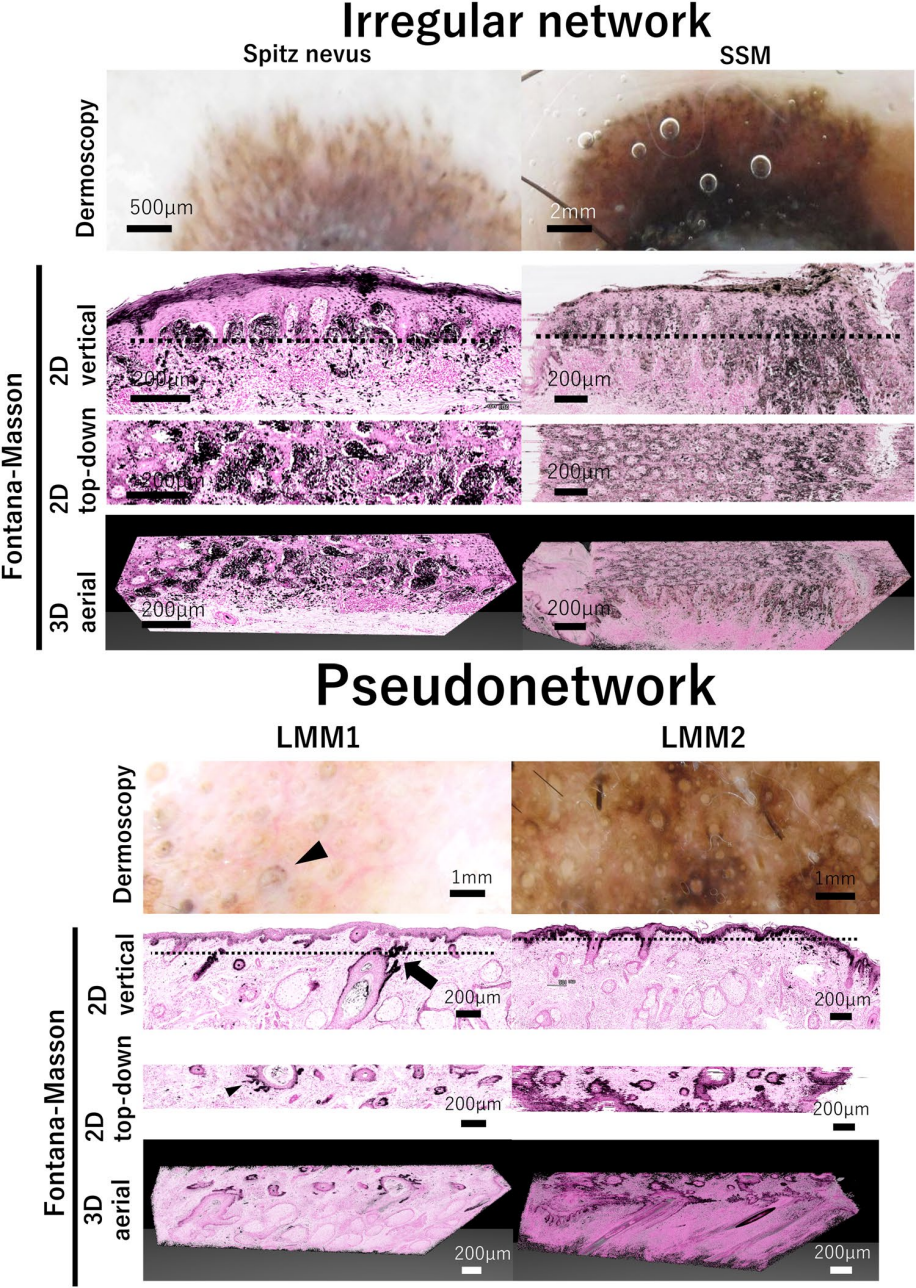
Irregular network and pseudonetwork structure and their correlations with histopathological morphology. Magnified dermoscopic image of irregular network, 2D vertical view image, reconstructed 2D top-down view image, and 3D aerial view image are shown in Spitz nevus, SSM, LMM1 and LMM2. Dotted line in vertical view image shows the level of depth in top-down view image. In the lower left panel, an arrowhead shows crescent structure in dermoscopy, and an arrow shows a projection of invading pigmented cells.

Histopathologically, reticular nevi are benign melanocytic proliferations with pigmented, thin, and elongated rete ridges. The network of rete ridges is expected to correspond to the reticular pattern in dermoscopy^13^. On the other hand, the irregular lines of an atypical network correspond to variation in the width, length, and spacing of the rete ridges, and to tendency to confluence of nests of pigmented cells^14^. In our study, a Spitz nevus showed irregular networks in dermoscopy (Fig. 2, upper panel). The irregular network had more variations in width and color than did the regular network. The reconstructed 2D top-down view images of the lesion showed that confluence of nests in the epidermal-dermal junction contributed to form an irregular network. In SSM1, we were able to observe irregular network on the fringe of the lesion (Fig. 2, upper right panel). This irregular network corresponded to the irregularly distributed pigmented cells in the rete ridges, as shown in the top-down view images.

### Pseudonetwork

Pseudonetwork and annular granular structures were the most frequent dermoscopic patterns among pigmented lesions of the face, irrespective of their benign or malignant nature^15^. Anatomically, the structure of facial skin has poor rete ridges, and instead, it has a large number of hair follicles. When pigmented cells exist in the epidermis, homogeneous pigmentation is disrupted by unpigmented follicular openings forming pseudonetwork^5^.

Dermoscopy of LMM1 and 2 on the face showed pseudonetwork (Fig. 2, lower panel). Moreover, there was a crescent-shaped pigmentation that rims one portion of the follicular opening (Fig. 2, lower panel; arrow head). It was asymmetric and semicircle-shaped, signet ring-like circle, or irregular circle with a variety of darkness. This structure is named hyperpigmented follicular openings and specific to early LMM^16^. 2D top-down view images and 3D aerial view images of LMM1 and LMM2 showed follicular invasions of melanoma cells, forming hyperpigmented follicular openings (Fig. 2, lower panel). These pigmented cells invaded the projection of adnexal structure from hair follicles (Fig. 2, lower panel; arrow).

Russo *et al*. suggested an interesting progression model for early LMM to the late LMM^4^. Namely, hyperpigmented follicular openings subsequently evolve into a granular–annular pattern, which corresponds to fine gray dots, globules and streaks around the follicles. Then, melanocytes surround and completely obliterate the follicular openings producing the dermoscopy feature of the rhomboidal structures.

### Dots

Dots are small, round structures of less than 0.1 mm in diameter with their color ranging from black, brown, to blue-gray depending on the depth and concentration of the melanin in the skin^17^. Black dots are often due to melanin pigment accumulation in the *stratum corneum*, but can also be due to vertical stacking of melanin within the epidermis or due to heavy aggregates of melanin in small clusters of melanocytes or keratinocytes located in the upper layers of the epidermis^4,17^.

**Figure 3:**
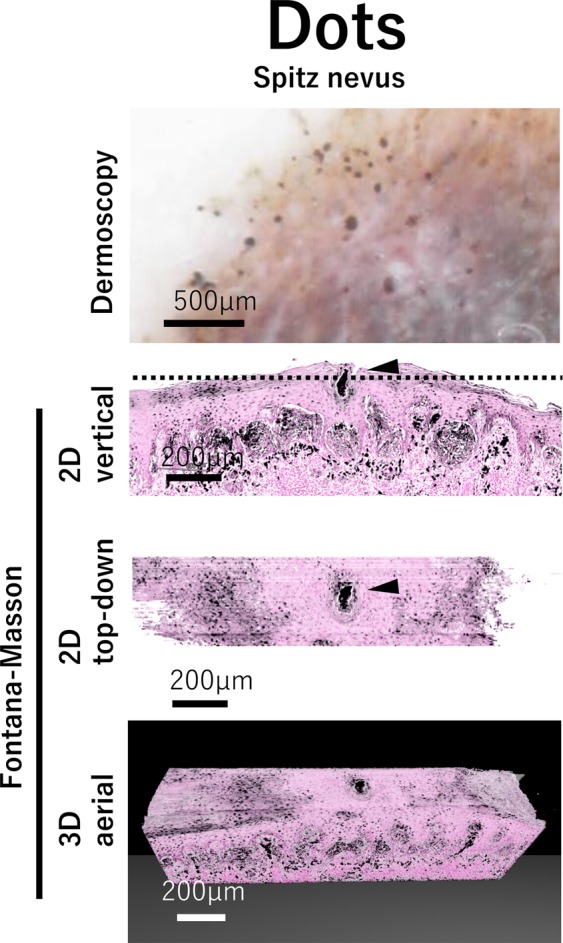
Dots structure and its correlation with histopathological morphology. Magnified dermoscopic image of dots, 2D vertical view image, reconstructed 2D top-down view image, and 3D aerial view image are shown in Spitz nevus. Dotted line in vertical image shows the level of depth in top-down image.

In our study, black, small, and clearly margined dots were observed in Spitz nevus (Fig. 3). 2D top-down view images and 3D aerial view images showed that these black dots were attributable to the densely pigmented aggregates of melanin in the cornified layer (Fig. 3). The diameters of small black dots were less than 0.1 mm.

### Globules

Globules are symmetrical, round to oval, well-demarcated structures with a diameter larger than 0.1 mm^18^. They are expected to correspond to nests of pigmented benign or malignant melanocytes situated in the lower epidermis, at the dermo-epidermal junction, or in the papillary dermis^18,19^. In our previous study, brown globules connected to the network were observed in dermoscopy. And the reconstructed 2D top-down view images and 3D aerial view images clearly showed that the nests of pigmented cells attached to the rete ridges in globular Nevus^9^.

### Cobble stone pattern

**Figure 4:**
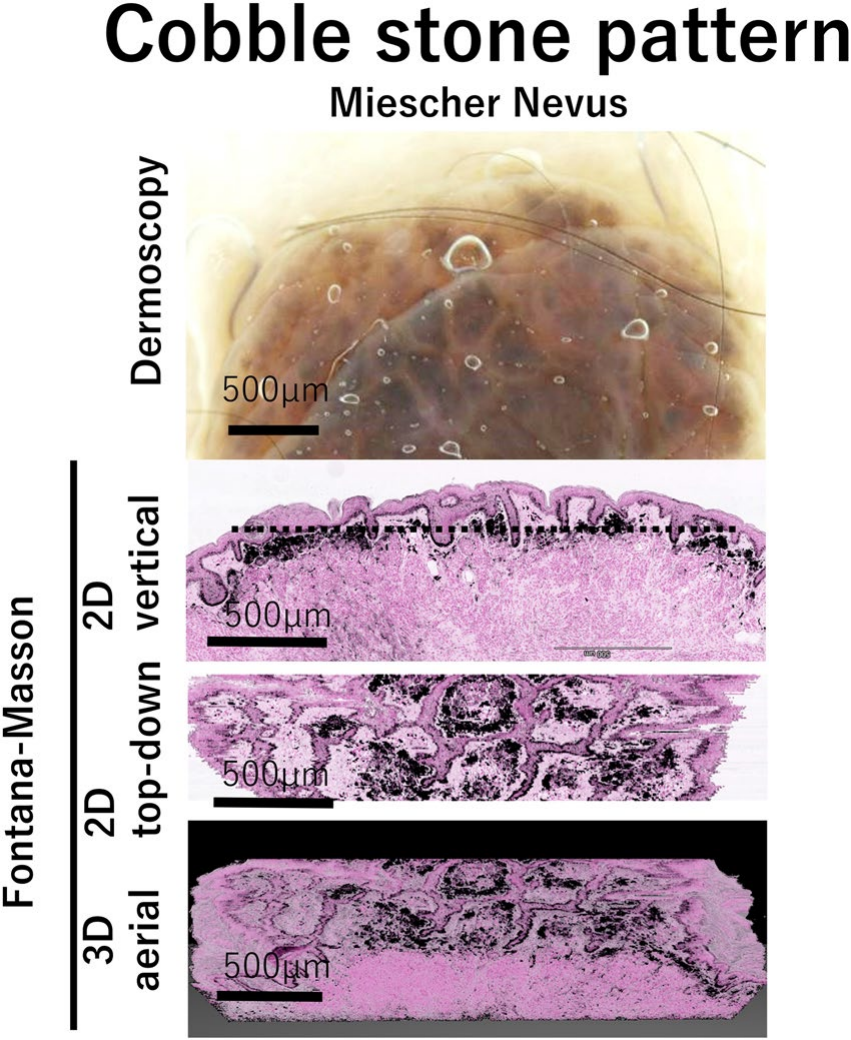
Cobble stone pattern structure and its correlation with histopathological morphology. As a variant of globules, cobble stone pattern is shown in Miescher nevus. Magnified dermoscopic image of cobble stone pattern, 2D vertical view image, reconstructed 2D top-down view image, and 3D aerial view image are shown in Miescher nevus. Dotted line in vertical view image shows the level of depth in top-down view image.

When globules are not perfectly round or oval, but have angulated corners, they are called cobblestone globules^20^. In our study, this pattern was observed in Miescher nevus, which was a dome-shaped nevus (Fig. 4). There was almost no report on the reason for appearance of this structure. While 2D vertical images exhibited only the nests of pigmented nevus cells on the upper dermis, reconstructed 2D top-down view images showed polygonal large network of rete ridges (Fig. 4). The area surrounded by rete ridges was filled with pigmented nevus cells, resulting in formation of the polygonally shaped nests, which could be recognized as the cobble-stone pattern in dermoscopy.

### Streak

**Figure 5:**
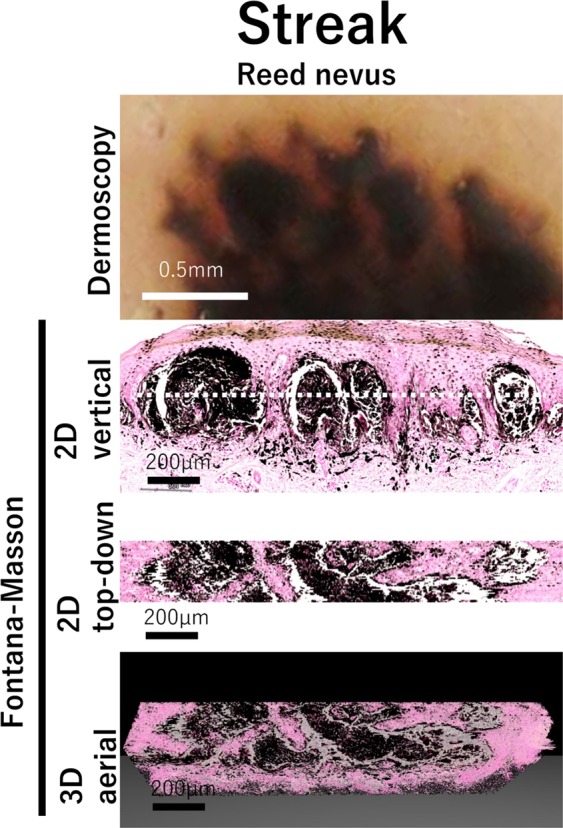
Streak structure and its correlation with histopathological morphology. Magnified dermoscopic image of streak, 2D vertical view image, reconstructed 2D top-down view image, and 3D aerial view image are shown in Reed nevus. Dotted line in vertical view image shows the level of depth in top-down image.

Streak, frequently seen in Reed nevus, is bulbous and often kinked or finger-like projections seen at the edge of a lesion. They are linear structures that may be observed throughout a lesion, but are more apparent in the periphery^19^. In our study, dermoscopy exhibited the streak in Reed nevus (Fig. 5). 2D vertical view image suggested enlarged nests of pigmented cells in the papillary dermis. 2D top-down view image and 3D aerial view image showed that these nests were connected linearly, suggesting that the linearly projected nests of pigmented cells construct the streak pattern.

### Homogeneously darkly pigmented lesion

**Figure 6:**
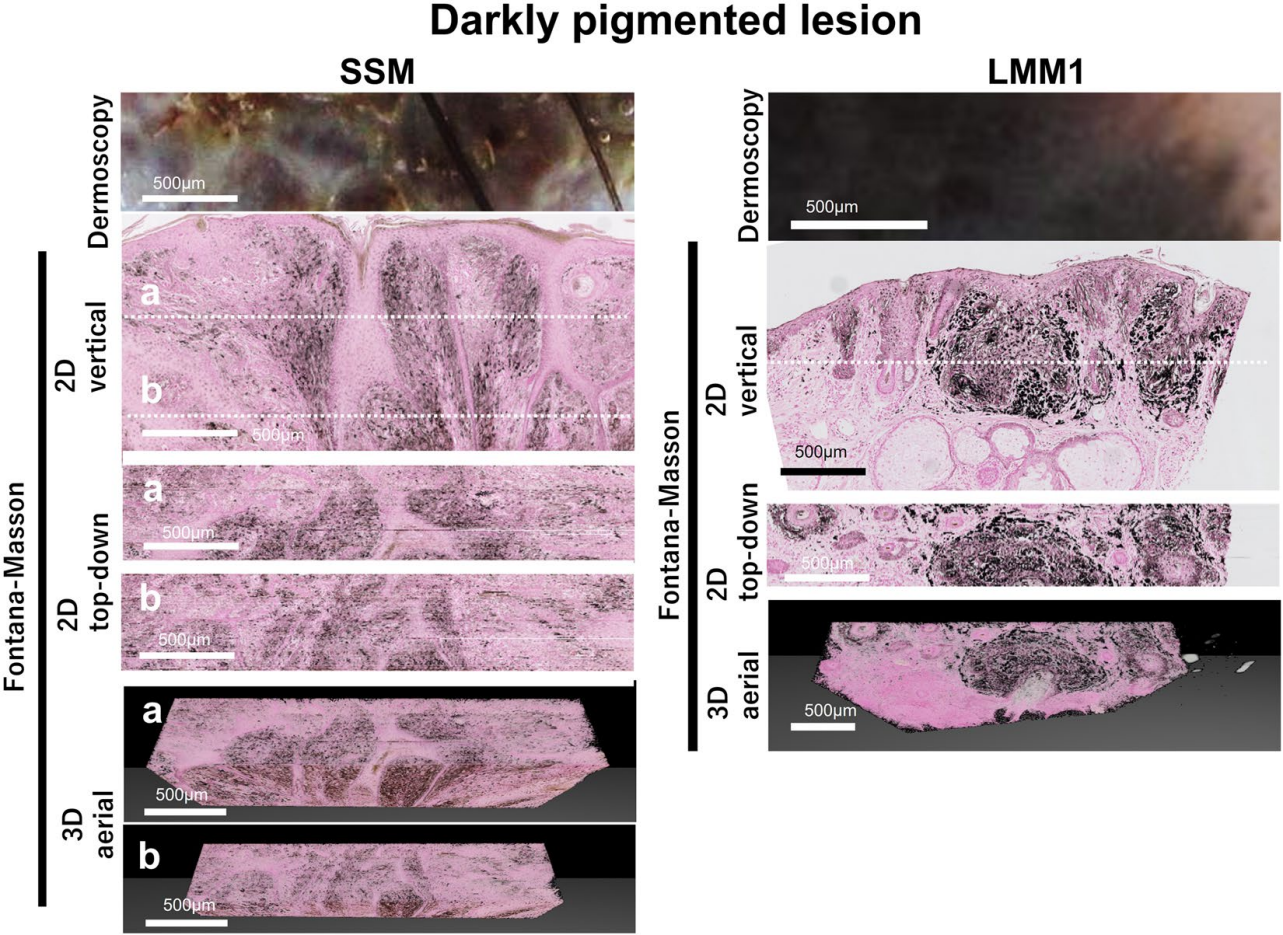
Homogeneously darkly pigmented structureless part and its correlation with histopathological morphology. Magnified dermoscopic image of the lesion, 2D vertical view image, reconstructed 2D top-down view image, and 3D aerial view image are shown in the homogenously darkly pigmented lesions of LMM1 and SSM. Dotted lines in vertical view image shows the level of depth in top-down images. In SSM, images with two different depth were shown as (**a**,**b**).

Our analysis unveiled some pathological structure which was not observed by dermoscopy. For example, the nodular lesion of SSM looks homogeneous and structureless in dermoscopy. On the other hand, reconstructed 2D horizontal top-down view revealed the dilated network of rete ridges. And the area surrounded by rete ridges was packed with nests of pigmented cells as we see in cobblestone pattern (Fig. 6, depth(a)). However, due to the irregularly reformatted network, the pattern was obscured (Fig. 6, depth(b)). And in the homogeneously pigmented lesion in LMM1, the reconstructed horizontal top-view image revealed the hair follicle densely surrounded by the nest of pigmented cells (Fig. 6).

## Conclusion

We reconstructed 2D horizontal top-down view images and 3D aerial view images from serial sections to explain the dermoscopic structures. Our study provides intuitive pathological explanation for the dermoscopic structure, including network, dots and globules, and streak.

## Supplementary information

Legends for supplementary videos

Supple1; Spitz; Irregular network & Dot

Supple2; Reed ;Streak

Supple3; Miescher; Cobble stone pattern

Supple4; LMM1; Pseudonetwork

Supple5; LMM1; Homogenously pigmented lesion

Supple6; LMM2; Pseudonetwork

Supple7; SSM; Irregular network

Supple8; SSM; Homogeneously pigmented lesion
