# Supplementary material for: An intuitive explanation of dermoscopic structures by digitally reconstructed pathological horizontal top-down view images
Source: Sci Rep. 2019 Dec 27;9:19875. doi: 10.1038/s41598-019-56522-8 (PMC6934765; doi:10.1038/s41598-019-56522-8)
Supplement: Supplementary file 1 — Legends for supplementary videos [file 41598_2019_56522_MOESM1_ESM.docx]

An intuitive explanation of dermoscopic structures by digitally reconstructed pathological horizontal top-down view images.

Akira KASUYA1, Masahiro AOSHIMA1, Kensuke FUKUCHI1, Takatoshi SHIMAUCHI1, Toshiharu FUJIYAMA1, Yoshiki TOKURA1

1Department of Dermatology, Hamamatsu University School of Medicine, 1-20-1 Handayama, Higashi-Ku, Hamamatsu 431-3192, Japan.

TEL+81-53-435-2303

FAX+81-53-435-2368

E-mail: casuakijo1@gmail.com

**Legends for supplementary videos**

Supple1; Spitz; Irregular network & Dot. A video of 3D aerial view of irregular network in Spitz nevus with various depth.

Supple2; Reed ;Streak. A video of 3D aerial view of streak in Reed nevus with various depth.

Supple3; Miescher; Cobble stone pattern. A video of 3D aerial view of cobble stone pattern in Miescher nevus with various depth.

Supple4; LMM1; Pseudonetwork. A video of 3D aerial view of pseudonetwork in LMM1 with various depth.

Supple5; LMM1; Homogenously pigmented lesion. A video of 3D aerial view of homogeneously darkly pigmented lesion in LMM1 with various depth.

Supple6; LMM2; Pseudonetwork. A video of 3D aerial view of pseudonetwork in LMM2 with various depth.

Supple7; SSM; Irregular network. A video of 3D aerial view of irregular network in SSM with various depth.

Supple8; SSM; Homogeneously pigmented lesion. A video of 3D aerial view of homogeneously darkly pigmented lesion in SSM with various depth.
